# Micromotor-mediated sperm constrictions for improved swimming performance

**DOI:** 10.1140/epje/s10189-021-00050-9

**Published:** 2021-05-11

**Authors:** Friedrich Striggow, Lidiia Nadporozhskaia, Benjamin M. Friedrich, Oliver G. Schmidt, Mariana Medina-Sánchez

**Affiliations:** 1 Institute for Integrative Nanosciences, Leibniz IFW Dresden e.V., Helmholtzstraße 20, 01069 Dresden, Germany; 2 Center for Advancing Electronics Dresden, TU Dresden, 01069 Dresden, Germany; 3 Cluster of Excellence ‘Physics of Life’, TU Dresden, 01307 Dresden, Germany; 4 School of Science, TU Dresden, 01062 Dresden, Germany; 5 Research Center for Materials, Architectures and Integration of Nanomembranes (MAIN), TU Chemnitz, Rosenbergstraße 6, 09126 Chemnitz, Germany

## Abstract

**Abstract:**

Sperm-driven micromotors, consisting of a single sperm cell captured in a microcap, utilize the strong propulsion generated by the flagellar beat of motile spermatozoa for locomotion. It enables the movement of such micromotors in biological media, while being steered remotely by means of an external magnetic field. The substantial decrease in swimming speed, caused by the additional hydrodynamic load of the microcap, limits the applicability of sperm-based micromotors. Therefore, to improve the performance of such micromotors, we first investigate the effects of additional cargo on the flagellar beat of spermatozoa. We designed two different kinds of microcaps, which each result in different load responses of the flagellar beat. As an additional design feature, we constrain rotational degrees of freedom of the cell’s motion by modifying the inner cavity of the cap. Particularly, cell rolling is substantially reduced by tightly locking the sperm head inside the microcap. Likewise, cell yawing is decreased by aligning the micromotors under an external static magnetic field. The observed differences in swimming speed of different micromotors are not so much a direct consequence of hydrodynamic effects, but rather stem from changes in flagellar bending waves, hence are an indirect effect. Our work serves as proof-of-principle that the optimal design of microcaps is key for the development of efficient sperm-driven micromotors.

**Graphic Abstract:**

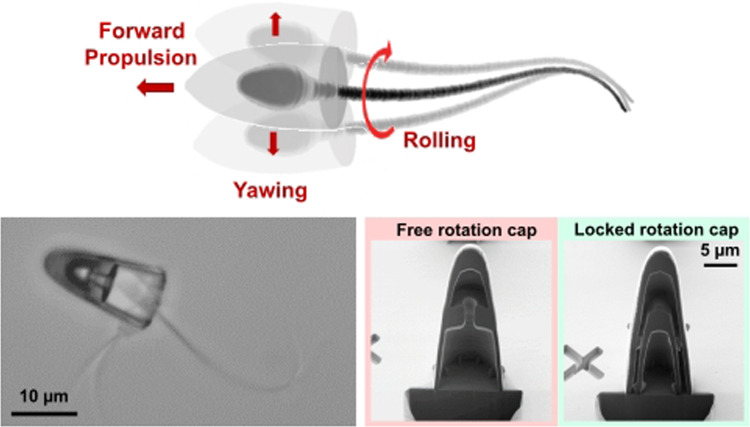

**Supplementary Information:**

The online version supplementary material available at 10.1140/epje/s10189-021-00050-9.

## Introduction

Microscale robotic swimmers are becoming a promising tool in biomedicine [1, 2] and environmental applications [3]. Due to their small size, these so-called microrobots or micromotors may allow new possibilities of noninvasive, targeted medical diagnosis [4], manipulation [5, 6] or drug delivery [7, 8]. A vast variety of different micromotors has been developed, using different propulsion mechanisms [9], including chemical reactions [10–13], physical actuation [14–17] or inclusion of motile cells or organisms [18–21]. The combination of biological components such as single [22–25] or multiple cells [26–28], and artificial components in biohybrid micromotors offers a number of design benefits. For instance, the strong propulsion of motile cells, *e.g.*, bacteria or sperm cells, can be utilized as a driving force to operate in complex biological fluids. Furthermore, specific abilities of the biological components can provide new functionalities, *e.g.*, sensing mechanisms such as chemotaxis or magnetotaxis [20, 29], tumor recognition and therapy [30, 31] or drug delivery by cell-to-cell biochemical interactions [24, 32].

An interesting approach to biohybrid micromotors is the use of sperm cells [33]. These highly motile cells offer a strong propulsion, and the unique ability to fertilize mature oocytes. This makes sperm-based micromotors an attractive option for applications in reproductive medicine [34]. The targeted transport and delivery of healthy spermatozoa could be utilized for a noninvasive, *in vivo *assisted fertilization, in order to circumvent obstacles of current In Vitro Fertilization (IVF) techniques, such as the relatively low success rate of embryo transfer to the uterus [35]. Since sperm cell are adapted to moving in the highly viscous and complex fluids of the female reproductive tract, they are ideal candidates to operate in biological environments [25]. The sperm head’s ability of fusing with other cells has furthermore opened up the possibility to use sperm-driven micromotors for the targeted delivery of drugs in the reproductive tract or even the circulatory system [24, 27, 36]. In this work, we employ micromotors for the transport of bovine sperm cells. For this purpose, single cells are captured in conical microcaps, which enable the magnetic control of the sperm-based micromotors. Optimizations of the design of such microcaps have focused on enabling the locomotion of micromotors in biological fluids such as oviduct fluid [25] or blood [27]. Still, upon coupling with such microstructures, cells are slowed down substantially, which limits the practical use of such sperm-driven micromotors in realistic settings. To further improve the performance of sperm-based micromotors, effects of added microstructures on the flagellar beat, and interactions between spermatozoa and their cargo should be considered.

**Fig. 1:**
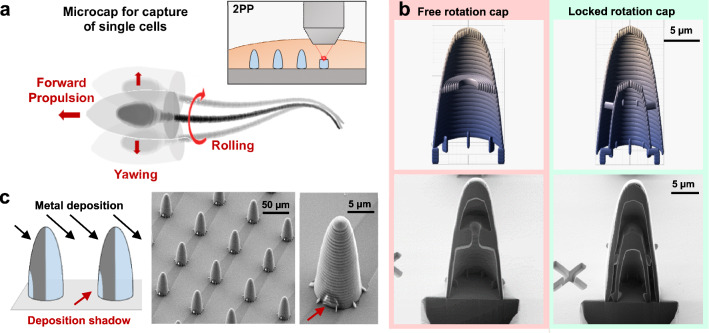
Fabrication of microcaps for sperm capture. **a** Schematic of sperm-driven micromotor showing the cell captured in a metal-coated microcap. Red arrows indicate typical movements caused by the sperms flagellar beat such as the forward propulsion, sideways yawing and rolling around its longitudinal axis. The inset depicts the process of two-photon photolithography (2PP). **b** Comparison of the designs (top) of the free rotation cap (FRC) and the locked rotation cap (LRC), and cross sections of the final structures prepared by a focused ion beam cut and imaged by SEM. **c** Schematic showing the process of metal deposition through electron beam evaporation, resulting in a deposition shadow on adjoining caps. The shadow is visible along the substrate and the lower part of the caps (red arrow) in the SEM images of the fabricated structures after metal coating

The efficient swimming of sperm cells (and many other motile cells) is induced by their flagellar beat. The flagellum is a long, slender filament extruding from the sperm head. It is built up of a ring of nine doublets of microtubules surrounding two single microtubules, which, in combination with a variety of different proteins, make up the axoneme [37, 38]. By hydrolysis of the chemical fuel adenosine triphosphate (ATP), dynein motor proteins generate shear forces that result in a sliding motion of neighboring microtubules, which leads to a bending deformation of the axoneme [39, 40]. A dynamic instability results in an oscillatory bending of the flagellum, with concomitant traveling bending wave propagating from the proximal to the distal end of the flagellum. Sperm cells do not simply swim straight, instead the sperm head usually describes a wiggling side-to-side motion around an averaged path, which may itself be curved or twisted. Due to the chiral architecture of the axoneme [38, 41], flagellar beat patterns are generally chiral and sperm cells typically move along chiral swimming paths [42, 43]. If flagellar bending waves are planar, but asymmetric in the plane of beating, resultant swimming paths will possess nonzero curvature, causing sperm cells to swim in circles [43]. An out-of-plane component of the flagellar beat, however, causes the cell to rotate around its longitudinal axis, corresponding to a *rolling* motion (see schematic in Fig. 1a). The resulting swimming path is straight but continuously twisted like a ribbon [43]. A combination of in-plane asymmetry and out-of-plane beating results in helical swimming paths along which the cell moves with continuous rolling on a path of constant curvature. Helical swimming can typically be observed for sperm cells moving in low viscosity media, whereas rolling of cells ceases at high viscosities [44]. If the rate of rolling becomes sufficiently fast, the radius of the helical path becomes very small and cells swim essentially as a twisted ribbon along a straight averaged path [45]. Additionally, as alluded above, on the fast timescale of the flagellar beat, the sperm head performs a wiggling motion around its averaged path as the sperm head balances forces from the beating flagellum [46]. This wiggling is characterized by a fast-oscillatory rotation in the plane of beating, referred to as *yawing*, which provides a convenient way to estimate the frequency of the flagellar beat.

Optimizing the design of sperm-based micromotors should thus address the effect of partially constraining the translational and rotational motion of the sperm cells by coupling them to artificial structures, as well as indirectly feedback on the flagellar beat of the sperm cells. In this work, we analyze, how the additional load of an artificial microstructure affects flagellar swimming and the efficiency of micromotors. Additionally, we investigate the influence of cell rolling and yawing by employing a dedicated cap design and applied magnetic field, respectively. In particular, two designs of microcaps are fabricated and used to capture single cells. In one structure, cells can roll freely, whereas they are rotationally locked in the second structure. A magnetic field is then applied, in order to constrain the yawing of cells, to investigate how swimming behavior changes once the micromotors are externally controlled. We analyze the effect of constraining these two degrees of freedom on the swimming performance and draw conclusions for the further optimization of sperm-driven micromotors.

## Results

### Fabrication of microcaps

We realized sperm-driven biohybrid micromotors by capturing single sperm cells in specifically designed microcaps (Fig. 1a). The flagellar beat provides the propulsion for the micromotor, whereas the cap allows for remote magnetic control.

Microcaps were fabricated using two-photon photolithography (2PP) of photoresist on silica substrates (Inset in Fig. 1a). This technique enables a precise fabrication of three-dimensional structures, with resolutions of up to 500 nm. Caps were fabricated in an upright manner in arrays of 7x7 structures. They have a conical shape with a rounded top which decreases hydrodynamic resistance and facilitates its movement through obstacles, *e.g.*, other cells [25]. Posts were introduced at the bottom of the structures to elevate the caps off the substrate, which ensures the removal of unpolymerized photoresist during the development. Additionally, the posts facilitate the mechanical release of caps from the substrate.

We fabricated two different designs of microcaps (Fig. 1b) to investigate the effect of constrained cell rolling and yawing on the motion of the micromotors: The Free Rotation Cap (FRC), in which a captured sperm cell can freely roll, and the Locked Rotation Cap (LRC), which limits the rolling of cells inside the cap. Both types of caps have the same outer dimensions, with a diameter of $9 \, \upmu \text {m}$ at the wider end and a length of $20 \, \upmu \text {m}$. The caps only differ in the designs of their insides. The FRC has a large circular cavity, allowing the sperm head to rotate freely inside the cap. The LRC has a narrow, flattened opening with a diameter of $7 \, \upmu \text {m}$ on the long axis and $5 \, \upmu \text {m}$ on the short axis at the widest point. Due to the flat shape of the sperm head, it only fits this cavity in a fixed position: once the cell is captured in the LRC, it is blocked from rotating inside the cap. A focused ion beam (FIB) cut was performed to prepare cross sections of both types of fabricated caps (see Fig. 1b, bottom). The different inner structures of both caps can be clearly distinguished: A large, round cavity in the FRC cap, and a narrow cavity in the LRC. Overall, the fabricated structures are in good agreement with the original designs.

Remote control of the moving micromotors can be achieved by a one-sided coating of iron, which causes the microcaps to align in an external magnetic field of 5 mT or 10 mT. A 10 nm layer of iron, enclosed by layers of titanium, is deposited by electron beam evaporation. Since the deposition happens at an angle, a part of the cap is shadowed from the deposition by its neighboring cap in the array (Fig. 1c). This deposition shadow can be used as an easily recognizable feature for measuring the micromotors rolling motion. The SEM images in Fig. 1c show an array of microcaps, with the shadowed area on the substrate surface, reaching up to the neighboring structure, clearly visible.

### Micromotor motion and flagellar beating

In order to fabricate motile sperm-driven micromotors, single bovine sperm cells were co-incubated with the microcaps. The captured cells were observed using a Zeiss Axio Observer, equipped with a phase-contrast filter. Briefly, the caps were mechanically released from their substrate and mixed with sperm cells in the sperm-specific medium SP-TALP. The medium is then transferred to a sample channel for observation (details in Materials and Methods). High framerate videos (500 Hz) were then recorded to visualize the flagellar beat and to analyze the movement of sperm-driven micromotors accurately.

**Fig. 2:**
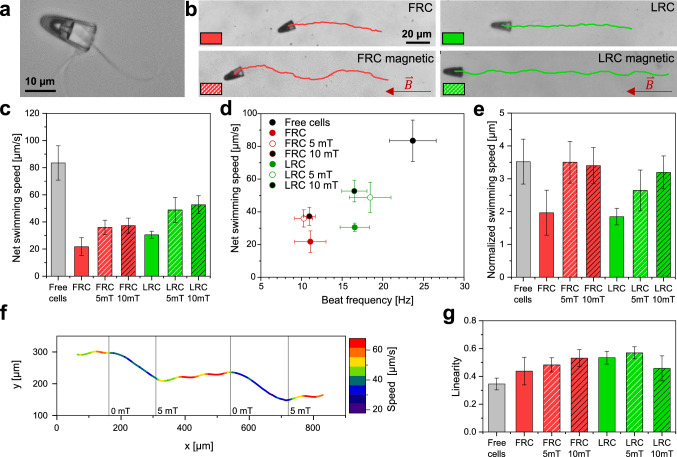
Motion of different sperm-driven micromotors. **a** A coupled micromotor imaged by brightfield optical microscopy. The sperm head can be seen inside the microcap with the flagellum protruding from the cap. A second cell can be seen underneath the microcap. **b** Representative trajectories of FRC and LRC micromotors swimming in sperm medium for a time of 4.5 s. The trajectories in the presence of a magnetic field of 5 mT are shown in the bottom row. **c** Net swimming speed of freely swimming sperm cells, FRC and LRC micromotors, with and without a magnetic field. **d** Net swimming speed *v* is plotted against flagellar beat frequency f of cells captured in different microcaps. **e** The normalized swimming speed, defined as the ratio *v*/*f*, of free-swimming cells and sperm-driven micromotors. **f** Trajectory of a FRC micromotor moving while the magnetic field (5 mT) is periodically switched on and off. **g** Linearity of cells and micromotors, defined as VSL/VCL. Error bars represent standard deviation

A coupled sperm-driven micromotor is shown in Fig. 2a. The structure of the FRC can be clearly seen, with only the sperm head captured in the cap and a free flagellum. Representative trajectories of micromotors with either FRC or LRC caps are shown in Fig. 2b (see Movie S1). After coupling with a sperm cell, micromotors are propelled forward by the flagellar beat of the sperm cells. The trajectory shows small sideways deviations, caused by an oscillatory yawing motion of the sperm head at the frequency *f* of the flagellar beat (details in Sect. 2.4). In the presence of a static magnetic field (bottom row), we additionally observe pronounced, sideways deviations at a slow frequency. We interpret these as a planar projection of helical swimming paths, where the magnetic caps keep a constant orientation angle relative to the external magnetic field. The different trajectory lengths in the various conditions are indicative of different swimming speeds.

We measured Curvilinear Velocity (VCL), Average Path Velocity (VAP) and Straight Line Velocity (VSL) of freely swimming cells and micromotors as commonly used in computer-assisted sperm analysis (CASA). We refer to VAP as the net swimming speed and use this quantity to evaluate the micromotors swimming performance. The net swimming speeds of sperm-driven micromotors are depicted in Fig. 2c. In comparison with freely swimming cells, cells captured in microcaps are substantially slowed down (for values, see Table 1). FRC and LRC micromotors are slowed down to 26 % and 37 % of free cell swimming speed, respectively. In the presence of a magnetic field, both types of micromotors swim faster than without the field, apparently independent of the strength of the applied field (at 5 mT and 10 mT): In case of the LRC in a 10 mT field ($52.7 \pm 6.6 \, \upmu \text {m}/\text {s}$), micromotors move at 63 % the speed of freely swimming sperm cells, which marks a substantial improvement in comparison with previous designs [25].

The net swimming speed *v* of micromotors depends in a non-trivial fashion on the waveform of flagellar bending waves, yet is independent of the viscosity of the surrounding fluid itself. The fact that *v* is independent from fluid viscosity, provided the frequency and the shape of flagella bending waves remains the same, is a direct consequence of the physics of microswimming at low Reynolds numbers, where inertia is negligible and the hydrodynamics of self-propulsion is governed by the linear Stokes equation. [47]. In this case, all hydrodynamic forces scale linearly with fluid viscosity. The instantaneous swimming speed is then determined by a force balance between two different hydrodynamic friction forces (namely a friction force associated with the shape change of the beating flagellum without any motion and a friction force associated with only motion but no shape change) [48]. As a consequence, the viscosity drops out in the computation of the swimming speed.

This theoretical fact does not contradict the experimental observation that measured swimming speeds *do* depend on fluid viscosity because any change of the viscosity of the surrounding fluid inevitably changes also the flagella beat pattern. Specifically, increasing fluid viscosity will increase the hydrodynamic friction forces acting on the beating flagellum and thus feedback on the molecular motors that drive its bending waves. Thus, both the frequency and the amplitude of flagellar bending waves are likely to decrease, resulting in a concomitant decrease in swimming speed. We emphasize that this dependence of swimming speed on fluid viscosity is a genuinely indirect effect that relates to the very mechanisms that orchestrate the flagella beat. It should not be surprising that this indirect dependence of swimming speed on hydrodynamic load is difficult to predict theoretically because it does not constitute a pure hydrodynamics problem anymore. Instead, modeling this flagella load response would require detailed knowledge on the precise mechanisms regulating the flagellar beat, which is a field of active research [39, 49].

A second consequence of the linearity of the Stokes equation is that the net swimming speed *v* scales proportional to the frequency *f* of the flagellar beat, provided no external forces act on the microswimmer. Rescaling the beat frequency by a constant factor simply rescales all flow fields and hydrodynamic friction forces by the same factor. Hence, the motion of the microswimmer, and in particular, its net swimming speed are rescaled as well. We thus have

1 $$\begin{aligned} v \sim f. \end{aligned}$$

To separate off this almost trivial dependence of net swimming speed on beat frequency, we report a normalized swimming speed *v*/*f* in Fig. 2e. The factor of proportionality between *v* and *f* in Equation (1) depends in a non-trivial way on the exact geometric shape of the flagellar beat. Analytical results are only available for very simple beat patterns and in the limit of small beat amplitudes [46]. In general, the net swimming speed has to be computed numerically for a given flagellar beat pattern, either by solving the Stokes equation or by employing one of various approximation schemes such as resistive force theory employed below. A particular feature of self-propulsion of non-symmetric swimmers is the coupling between different degrees of motion, e.g., translational and rotational degrees of freedom.

Specifically, we first introduce the (time-dependent) constraining force **F** and the constraining torque **M** that would be needed to restrain a microswimmer from moving, while the flagellum continues to beat normally. Second, we introduce the grand hydrodynamic friction matrix $\varvec{\Gamma }$ of a rigid microswimmer that does not change its shape: this 6x6 matrix $\varvec{\Gamma }$ relates the instantaneous translational velocity vector **v** and the instantaneous rotational velocity vector $\varvec{{\upomega }}$ at which such a microswimmer is dragged through the fluid to the force $\mathbf{F }_{\mathrm {drag}}$ and $\mathbf{M }_{\mathrm {drag}}$ exerted by this microswimmer on the surrounding fluid as

2 $$\begin{aligned}{}[\mathbf{F }_{\mathrm {drag}}, \mathbf{M }_{\mathrm {drag}}] = \varvec{\Gamma } [\mathbf{v} , \varvec{{\upomega }}]. \end{aligned}$$

A self-propelled microswimmer free from external forces and torques does not exert any net force or torque on the surrounding fluid. Thus, for such a self-propelled microswimmer, we must have $\mathbf{F }_{\mathrm {drag}} + \mathbf{F} = \mathbf{0}$ as well as $\mathbf{M }_{\mathrm {drag}} + \mathbf{M} = \mathbf{0}$ at any instance of time. This force and torque balance provides 6 linear equations, which uniquely determine the 6 components of the instantaneous translational velocity vector **v** and the instantaneous rotational velocity vector $\varvec{{\upomega }}$. Using **v** and $\varvec{{\upomega }}$ to integrate the trajectory of the microswimmer over a full beat cycle allows to finally determine the net swimming speed *v*. By coupling a sperm cell to a microcap, and thus increasing the cargo of this microswimmer, the entries of its hydrodynamic friction matrix will increase. This results in smaller instantaneous motion, and in general a reduced net swimming speed.

Yet, most importantly, the presence of the microcap can feed back on the generation of flagellar bending waves themselves, and hence change the flagellar beat frequency *f* in Eq. (1).

We therefore measured the flagellar beat frequency *f* of captured sperm cells, by analyzing high-speed video microscopy recordings of their flagellar beat, using the ImageJ plugin SpermQ [50]. Representative kymographs of the flagellar curvature are shown in Fig. S1 in supporting information. The swimming speeds of micromotors, in dependence of their particular flagellar beat frequency, are depicted in Fig. 2d. In comparison with freely swimming cells ($f = 23.7 \pm 2.9 \, \hbox {Hz}$), captured cells show a reduced beat frequency (see Table 1). Upon capture in the FRC and LRC, the beat frequency is reduced to 47% and 70% of the reference value of free cells, respectively.

This represents a flagellar load response, in which the speed (and possibly shape) of the flagellar beat depend on the hydrodynamic forces acting on the flagellum. This load response is consistent with previous observations of sperm cells swimming in high-viscosity medium, or ciliated microswimmers exposed to external flows [51, 52], and an expected feature of shape-changing active microswimmers in general [53]. Similarly, reduced frequencies of the flagellar beat of sperm cells have been reported for cells tethered on a surface [39].

Overall, swimming speed scales approximately with the beat frequency *f*, as suggested by Eq. (1). Interestingly, the reduction of *f* is stronger for sperm cells captured in the FRC. Deviations from a perfect linear proportionality between swimming speed *v* and beat frequency *f* suggest that also the geometry of flagellar swimming changes. Intriguingly, this effect does not depend simply on the effective hydrodynamic load of the microcap as suggested by a simple superposition principle. In fact, the hydrodynamic friction coefficients of the microcaps should be essentially identical for the different designs FRC and LRC. In contrast, the ratio *v*/*f* is different for the two designs and in particular depends on the presence of an external magnetic field: In the presence of the magnetic field, beat frequencies do not change significantly (FRC/FRC 5 mT: $p = 0.41$, LRC/LRC 5 mT: $p = 0.13$), whereas swimming speeds are increased for both caps (FRC/FRC 5 mT: $p = 5.36 \, 10^{-5}$, LRC/LRC 5 mT: $p = 1.17 \, 10^{-4}$).

**Table 1:** Swimming speed, beat frequencies and rolling frequencies of cells and caps in different conditions

	Free cells	FRC	FRC 5 mT	FRC 10 mT	LRC	LRC 5 mT	LRC 10 mT
$v [\upmu \text {m}/\text {s}]$	$83.5 \pm 12.6$ (n = 52)	$21.8 \pm 6.6$ (n = 10)	$36.0 \pm 5.3$ (n = 10)	$37.3 \pm 5.5$ (n = 10)	$30.6 \pm 2.5$ (n = 10)	$48.8 \pm 9.3$ (n = 10)	$52.7 \pm 6.6$ (n = 9)
*f* [Hz]	$23.7 \pm 2.9$ (n = 20)	$11.1 \pm 1.9$ (n = 6)	$10.3 \pm 1.1$ (n = 5)	$11.0 \pm 0.7$ (n = 7)	$16.6 \pm 1.8$ (n = 6)	$18.5 \pm 2.6$ (n = 8)	$16.5 \pm 1.6$ (n = 7)
$v/f [\upmu \text {m}]$	$3.5 \pm 0.7$	$2.0 \pm 0.7$	$3.5 \pm 0.6$	$3.4 \pm 0.6$	$1.8 \pm 0.3$	$2.6 \pm 0.6$	$3.2 \pm 0.5$
LIN	$0.35 \pm 0.04$ (n = 50)	$0.44 \pm 0.1$ (n = 10)	$0.48 \pm 0.05$ (n = 10)	$0.53 \pm 0.06$ (n = 10)	$0.53 \pm 0.05$ (n = 10)	$0.57 \pm 0.04$ (n = 10)	$0.46 \pm 0.09$ (n = 7)
$f_{sp}$ [Hz]	$6.3 \pm 1.4$ (n = 52)	$6.1 \pm 1.0$ (n = 10)	$5.7 \pm 1.0$ (n = 10)	$6.2 \pm 0.8$ (n = 10)	$1.3 \pm 0.3$ (n = 10)	$1.3 \pm 0.5$ (n = 10)	$1.8 \pm 0.5$ (n = 9)
$f_{mc}$ [Hz]	N.A.	$0.4 \pm 0.1$ (n = 20)	$0.3 \pm 0.1$ (n = 20)	$0.5 \pm 0.2$ (n = 20)	$1.1 \pm 0.2$ (n = 21)	$1.4 \pm 0.7$ (n = 20)	$1.5 \pm 0.4$ (n = 14)
$f_{sp}-f_{mc}$ [Hz]	N.A.	$5.7 \pm 1.0$	$5.4 \pm 1.0$	$5.7 \pm 0.9$	$0.3 \pm 0.4$	$-0.1 \pm 0.8$	$0.3 \pm 0.6$

The geometric ratio *v*/*f* ratio is reduced to similar values for cells captured in the FRC ($2.0 \pm 0.7 \, \upmu \text {m}$) and LRC ($1.8 \pm 0.3 \, \upmu \text {m}$), when compared to freely moving cells ($3.6 \pm 0.8 \, \upmu \text {m}$). Remarkably, it increases in the presence of a magnetic field, reaching almost the value of freely swimming cells for FRC micromotors.

Figure 2f (and Fig. S2) shows the trajectory of a FRC micromotor moving in a magnetic field of 5 mT that is periodically switched on and off, with the swimming speed represented by the color coding. As soon as the field is turned on, the micromotor changes orientation and its speed increases. It additionally starts moving on a helical path that is projected as a wave-shape trajectory. Measuring the swimming speed along this trajectory results in fluctuations of speed since the out-of-plane component of the helical path is not considered. When the field is switched off again, speed decreases and the micromotor moves along a smoother path.

In addition to swimming speed, we measured the linearity of swimming paths (Fig. 2f), which in CASA is commonly defined as $\hbox {LIN} = \hbox {VSL/VCL}$. The trajectories of freely swimming cells have a mean linearity of $0.35 \pm 0.04$. This value is increased for cells captured in both types of microcaps. The additional load of the microstructure likely reduces the sideways movements of the sperm head and hence the curvilinear velocity VCL, resulting in a higher linearity. Application of the magnetic field further increases linearity.

These results highlight the intricate trade-off choices of optimizing the design of sperm-based micromotors. Coupling to a microcap changes the hydrodynamics of swimming (incorporated in the geometric ratio *v*/*f*), yet also decreases the frequency *f* of the flagellar beat, which both determine net swimming speed *v*.

### Rolling of sperm cells and micromotors

The microcap partially constrains rotational motion of the sperm head. It is thus an important question to ask how microswimming changes if we impose a constraint on some, but not all degrees of freedom, e.g., if we would constrain only ‘yawing.’ For symmetric swimmers such as spheres, the grand hydrodynamic friction matrix is diagonal. Yet, for non-symmetric swimmers, the off-diagonal entries of this matrix will in general be nonzero, representing a coupling between the different degrees of freedom. If we impose a constraint on some degrees of freedom of the microswimmer, we have to cross out the corresponding rows and columns from the 6x6 matrix $\varvec{\Gamma }$ of friction coefficients as well as cross out the corresponding components of the 6-component vector Eq. (2), leaving a linear equation for the remaining degrees of freedom. Depending on the off-diagonal entries of the grand hydrodynamic friction matrix $\varvec{\Gamma }$, this may actually increase the net swimming speed.

To investigate the effect of constrained rolling on the performance of micromotors, we quantified both the rolling of cells inside the caps, as well as the rolling of the caps.

**Fig. 3:**
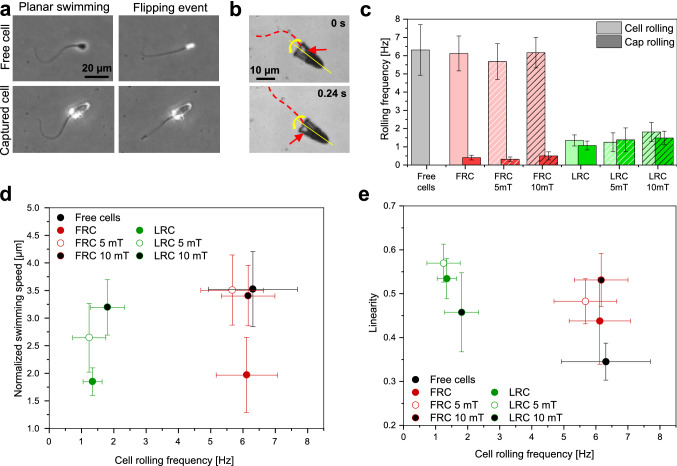
Rolling of freely swimming cells, FRC and LRC micromotors. **a** Rolling of sperm cells. Both freely swimming and captured cells roll around their long axis, with alternating periods of effectively planar swimming and rapid flipping events during which the flagellum appears almost straight (with flagellar bending only perpendicular to the imaging plane). **b** The microcap rotates around its symmetry axis. Rolling of the cap can be monitored by observing the periodic motion of the deposition shadow (red arrow) on the cap. Shown are brightfield images of FRC micromotors. **c** Rolling frequencies of cells inside the FRC and the LRC, as well as rolling frequencies of the caps themselves. **d** Normalized swimming speeds *v/f* of micromotors with either FRC or LRC caps plotted against the rolling frequency $f_{sp}$ of the (captured) sperm cells (with and without magnetic field). **e** Linearity of cell swimming trajectories plotted against the cell rolling frequency. Error bars represent standard deviation

Rolling of sperm cells can easily be observed in a microscope using a phase-contrast filter (see details in Sect. 5.5, Movie S3). The cell head and flagellum appear uniformly black when the flat sperm head is oriented parallel to the focal plane (Fig. 3a, ‘planar swimming’). Since the flagellar shape is approximately planar, the flagellum is in focus during this phase. During rolling, the cell flips from one side to the other, corresponding to a ‘flipping event’ during which the head briefly appears bright. The periodic changes in brightness of the head are commonly used to measure the rolling frequency of freely swimming sperm cells [54–56]. The rolling of sperm cells captured in microcaps cannot be observed as easily due to the bright appearance of the cap in phase-contrast imaging, which obscures the signal of the sperm head. Alternatively, the planar swimming state and the flipping state of cells can be distinguished by observing the shape of the flagellum (Movie S4). During planar swimming, the flagellar wave shape can be clearly seen. When the cell flips from one side to the other, the flagellum follows and at the moment of flipping, the flagellum bends perpendicular to the focal plane, resulting in an almost straight two-dimensional projection of the out-of-plane flagellar shape. These repeated events of low flagellar curvature can be tracked and used to determine the frequency $f_{sp}$ at which cells roll inside the microcap.

The rolling frequency of caps was measured separately to verify that cells can roll freely in the FRC and cells are rotationally locked in the LRC. The deposited metal layer (see Fig. 1d) is used to visualize and track rolling of the caps. While the polymeric microcap appears mostly transparent in brightfield optical microscopy, even thin metal films are opaque and can therefore be used to monitor rolling. Furthermore, the deposition shadow provides an easily recognizable feature that moves around the circumference of the cap during rolling (Fig. 3b & Movie S5). This feature appears periodically twice for every full rotation of the microcap and is used to measure the rolling frequency $f_{mc}$ of the micromotor. The motion of the deposition shadow additionally shows that both types of caps, and therefore also the captured sperm cells, are undergoing continuous $360^{\circ }$ rotation (Movie S5), in accordance with early observations of bull sperm rolling [55]. Similar observations have been reported for the rolling of human sperm [56], whereas mouse sperm have been reported to rotate in alternating directions [57, 58].

The comparison between cell rolling frequency ($f_{sp}$) and the microcap rolling frequency ($f_{mc}$) of different samples is shown in Fig. 3c. The corresponding rolling frequencies and swimming speeds are summarized in Table 1. Freely swimming sperm cells from all samples roll at a mean frequency of $6.3 \pm 1.4 \, \hbox {Hz}$. The rotation of cells, which are captured in the FRCs does not slow down significantly ($f_{sp} = 6.1 \pm 1.0 \, \hbox {Hz}$, $p = 0.58$), even though a slow rotation is induced in the moving cap ($f_{mc} = 0.4 \pm 0.1 \, \hbox {Hz}$). These numbers imply that cells roll at a frequency of $5.7 \pm 1.0 \, \hbox {Hz}$ relative to the cap, demonstrating that cells can roll almost freely inside the FRC as intended by design. Application of a static magnetic field does not affect this behavior, independent of field strength. In contrast, a substantial decrease in $f_{sp}$ can be observed for the sperm cells captured inside the LRCs ($f_{sp} = 1.4 \pm 0.3 \, \hbox {Hz}$). The rotation frequency $f_{mc}$ of the micromotors is increased for LRC compared to the FRCs and reaches a value close to the rotation frequency of the cells themselves ($f_{mc} = 1.1 \pm 0.2 \, \hbox {Hz}$). Thus, in the LRC, $f_{sp}$ and $f_{mc}$ are essentially the same (see Table 1). This shows that sperm cells are rotationally locked inside the LRC in an effective manner, and the torque generated by the chiral flagellar beat causes a counter-rotation of the caps (albeit with reduced rolling frequency compared to the case of freely swimming cells due to the increased hydrodynamic resistance of the cap). Again, we observe a similar behavior in the presence of the magnetic field for the LRC design. In summary, both the FRC and the LRC design perform as intended, with the FRC enabling the free rotation of the cell, while the LRC constrains this rolling motion.

Figure 3d shows the normalized swimming speed *v*/*f* in dependence of the cell rolling frequency $f_{sp}$. Notably, the values of *v*/*f* are similar for both FRC and LRC micromotors, irrespective of their different cell rolling frequencies. In the presence of an external magnetic field, we observe an increase in the normalized swimming speed for both designs, yet the cell rolling frequency barely changes. The linearity of swimming paths in dependence of the cell rolling frequency $f_{sp}$ is depicted in Fig. 3e. While there are changes in linearity within a specific microcap design (FRC or LRC) upon application of the magnetic field, this appears to be independent of the cell rolling frequency, as $f_{sp}$ remains basically unaffected by the increased field strength within one group.

This suggests that constraining the rolling motion of sperm cells, if at all, has only a negligibly small direct effect on the swimming efficiency of the biohybrid micromotor.

### Yawing of micromotors

**Fig. 4:**
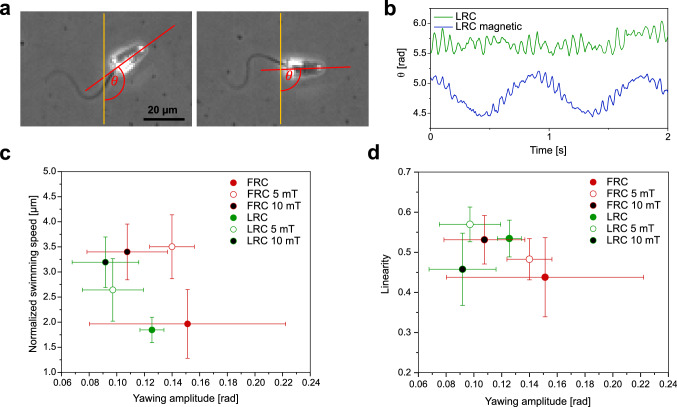
Yawing of micromotors **a** The orientation of the microcap is quantified by measuring the angle $\theta$ between the central axis of the cap (red line) and a fixed reference (orange line). **b** Monitoring the angle $\theta$ over time reveals periodic changes in orientations, corresponding to a yawing motion of the cap. **c** The normalized *swimming speed, v/f, is plotted against the* amplitude of the yawing motion. **d** The linearity of micromotor swimming paths is negatively correlated with yawing amplitude. Error bars represent standard deviation

In addition to rolling, the flagellar beat of a sperm cell typically induces an oscillatory yawing motion of the sperm head. The resultant sideways motion can also be observed in sperm-driven micromotors. By tracking the orientation of the microcap in reference to a fixed vector, the yawing angle ${{\theta }}$ can be determined (Fig. 4a).

Monitoring the orientation over time reveals periodic changes of the angle $\theta$ (Fig. 4b). In the presence of a magnetic field, we observe a prominent low-frequency component (blue curve), which is caused by the continuous alignment of the cap with the field, as explained in Sect. 2.2. In all cases, we additionally observe a high-frequency component (blue and green curve), which can be directly attributed to the flagellar beat. In fact, the frequency of this fast yawing motion provides an independent measurement of the beat frequency *f*, which is in agreement with the beat frequencies determined from flagellar tracking shown in Fig. 2d (Table S1 in supporting information). Measuring the amplitude of these periodic changes in microcap orientation, allows us to compare the effect of sperm capture in different caps on the characteristic yawing motion (Fig. 4c). Upon application of the magnetic field, the yawing amplitude of LRC micromotors slightly decreases from $0.13 \pm 0.01$ rad to $0.11 \pm 0.05$ rad ($p = 0.014$). The alignment of the cap with the field constrains the in-plane rotation of the cap, and thus sperm yawing. Due to the large variance in the yawing amplitude of FRC micromotors, no conclusion can be drawn about the effect of the magnetic field on the swimming speed in this case.

Figure 4d shows that the yawing amplitude decreases with increasing strength of the external magnetic field. This is seemingly correlated with an increasing linearity of the micromotors swimming paths. For FRC micromotors, an increase in magnetic field strength from 5 to 10 mT results in an increased linearity. The stronger alignment at higher field strength likely leads to a reduced yawing motion, which, in turn, decreases the length of the curvilinear path of the micromotor, resulting in a lower curvilinear velocity. Since linearity is defined as the ratio VCL/VSL, this reduction of VCL results in an increased linearity.

While we do not observe a direct effect of constrained yawing motion on the normalized swimming speed of sperm-driven micromotors, linearity increases when yawing is reduced by the applied magnetic field. This observation can partly explain the improved performance of FRC and LRC micromotors moving in the presence of the external field.

### Modeling/simulations

To gain basic insight into flagellar swimming under load, we numerically computed sperm swimming speeds using resistive force theory applied to a prototypical three-dimensional beat pattern previously determined for sea urchin spermatozoa [43].

In short, we considered a prototypical three-dimensional flagellar beat pattern, where the torsion ${\uptau }_{0}$ of the flagellar centerline is constant, while its curvature ${\upkappa }(s,t)$ is given by a traveling wave

$$\begin{aligned} {\upkappa }(s,t) = {\upkappa }_{0} + A \, \cos (2 {\uppi }(s/{\uplambda }- f t)). \end{aligned}$$

with mean curvature ${\upkappa }_{0}$, amplitude *A*, wavelength ${\uplambda }$ and frequency *f*, where $0{<} s {<} L$ denotes arc length along the flagellum (with $s=$0 fixed at the center of the spheroidal head) and time *t*. Parameters: ${\upkappa }_{0} = 0.03507 \, \upmu {\hbox {m}}^{{-}1}$, ${\uptau }_{0} = 0.00477 \, \upmu {\hbox {m}}^{{-}1}$, ${ A} = 0.16 \, \upmu {\hbox {m}}^{{-}1}$, ${\uplambda }=29.6 \, \upmu \hbox {m}$, $f= 43.5 \, \hbox {Hz}$, $L= 41 \, \upmu \hbox {m}$ [43].

For the implementation of resistive force theory [48], we computed the hydrodynamic friction forces acting on the micromotor as

$$\begin{aligned} \mathbf{F }= & {} \mathbf{F }_{\mathrm{cap}} + \int _0^L \, \mathrm{{d}}s \, \xi _{\parallel } [{\dot{\mathbf{r }}} (s,t) \cdot \mathbf{t } (s,t)] \mathbf{t } (s,t) \\&+ \xi _{\perp } ({\dot{\mathbf{r }}} (s,t) - [{\dot{\mathbf{r }}} (s,t) \cdot \mathbf{t } (s,t)] \mathbf{t } (s,t)), \end{aligned}$$

where $\mathbf{F }_{\mathrm {cap}}$ is the friction force of the cap (or sperm head for free swimming cells), $\dot{\mathbf {r}} (s,t)$ is the instantaneous velocity of the flagellar centerline $\mathbf {r} (s,t)$ at arc length position *s* and time *t*, $\mathbf{t } (s, t) = \partial \mathbf{r } (s, t)/\partial s$ is the local tangent vector, and $\xi _{\parallel }$ and $\xi _{\perp }$ are hydrodynamic friction coefficients for parallel and normal motion, respectively, with $\xi _{\parallel } = 0.69 \, \hbox {pN ms}/\upmu {\hbox {m}}^{2}$ and $\xi _{\perp }/\xi _{\parallel }= 1.81$ for dynamic fluid viscosity 0.7 mPa s [46]. The cap was modeled as a spheroid with semi-axes 10 $\mu$m $\times$ 5 $\mu$m $\times$ 5 $\mu$m (rigidly attached to the flagellum, dimensions comparable to the microcaps used in this study) and the Perrin formulas used to compute the hydrodynamic drag. Of note, in the low-Reynolds number limit, the hydrodynamic drag of an object depends only weakly on its shape, but only on its dimensions. A smaller spheroid with semi-axes 5 $\mu$m $\times$ 2.5 $\mu$m $\times$ 2.5 $\mu$m was used to model the sperm head of free swimming sperm cells. The computation of torques **M** is analogous.

By computing separately the forces $\mathbf{F }_{\mathrm {drag}}$ and torques $\mathbf{M }_{\mathrm {drag}}$ for a rigid body corresponding to either a translation along one of the coordinate axes of the material frame of the micromotor with unit speed, or a rotation around one of these axes with unit rotational speed, we obtain all components of the 6x6 grand hydrodynamic friction matrix ${\varvec{\Gamma }}$ [59]. Similarly, we compute the force **F** and torque **M** corresponding to the active shape change of the flagellum for a clamped cell whose head is constrained from translation and rotation. Thereby, we obtain a linear equation system for the instantaneous velocity vector **v**(*t*) and rotational velocity vector ${\varvec{{\upomega }}} (t)$ of the micromotors (relative to the material frame of the swimmer). Integration in time (using a simple Euler scheme) yields the swimming trajectory, from which we can read off the net swimming speed *v*.

We find that the normalized net swimming speed *v*/*f* is reduced to 51.5% for micromotors with a cap compared to free swimming sperm cells. This reduction in speed is similar to the experimentally observed reduction of normalized swimming speed of 55% and 50% for FRC and LRC micromotors compared to free swimming cells, respectively (see Table 1).

If we additionally constrain yawing motion of the micromotor in the computations, we find a reduction of swimming speed of only 53.9%: thus, constraining yawing helps micromotors to swim a bit faster, but not much. Constraining all rotational motion has a similar effect (speed reduction: 54.5%), whereas constraining rolling has virtually no effect (speed reduction: 51.6%).

In conclusion, the observed reduction of normalized swimming speed *v*/*f* of micromotors compared to free swimming cells can likely be attributed to the presence of an additional hydrodynamic load. However, our observation that an external magnetic field can increase again the normalized speed *v*/*f* cannot be fully explained as a direct consequence of hydrodynamic effects: although the application of an external magnetic field constrains yawing motion, we predict that such a constraint should have only a small effect on swimming speed. Instead, we expect that the shape of the flagella beat changed as a consequence of constrained yawing.

## Discussion

To design efficient sperm-based micromotors, the interactions between the cell and any artificial structure should be well understood. In the present work, we focus on micromotors, which are used for the transport of single sperm cells. Tubular microstructures, fabricated either by rolling up nanomembranes [33], maskless lithography [60] or two-photon photolithography [24, 25], have been used for this purpose. Even though coupling of sperm cells to such artificial components results in a poor swimming performance when compared to freely swimming cells, it offers several advantages. For instance, through the integration of magnetic materials as iron or nickel, such microstructures coupled to sperm cells can easily be controlled by an external magnetic field. Magnetic fields are often the method of choice to remotely control micromotors, as they enable fast and precise control, penetrate tissues, are biocompatible, and can therefore be used even in *in vivo* scenarios [61]. Furthermore, the results shown in the present work point to a positive effect of static magnetic fields on the effective swimming of sperm-driven micromotors. The presence of an external alignment field substantially increases net swimming speed, while it concomitantly reduces the yawing motion of the micromotors which results in a higher linearity. By optimizing the design of the artificial microstructure, the swimming performance of micromotors can be improved in order to facilitate swimming in complex biological fluids [25].

While constraining non-essential motion such as yawing can indeed increase net swimming speed, this effect is weak and can only partially explain the observed increase in swimming speed. Rather, the increase in swimming speed is most likely related to a favorable change of the flagellar beat pattern due to a feedback of the additional cargo on the internal force generating mechanism of the flagellum. Previous studies, where translational and rotational motion of sperm cells were constrained, included, *e.g.*, fixation of sperm heads in micropipettes [62, 63], attachment to glass surfaces [39] or capture of cells in optical traps [56]. The reported effects of mechanically constrained cell translation and rolling on flagellar beat frequency have been inconsistent, with some studies showing no effect while others report a decrease in beat frequency by one-third [39, 63]. We observe a decrease in frequency *f* by $\sim$ 50% for the FRC and $\sim$ 30% for the LRC, independent of the applied magnetic field. Depending on the design of the microstructure that they are captured in, cells fall into two distinct categories: fast rolling cells with a slower flagellar beat (FRC) or slow rolling cells with a faster flagellar beat (LRC) (see Fig. S3 in supporting information). While we do not have a mechanistic explanation, why the beat frequency in FRC micromotors is smaller compared to LRC micromotors, we speculate that constraining cell rolling has a particularly strong effect on bend initiation at the proximal tip of the flagellum. This peculiar behavior is worth further investigation and may be informative in refining existing theories of motor control underlying flagellar bending waves [39, 49, 64, 65].

Besides hydrodynamic and biophysical considerations, mechanoresponses of sperm cells may contribute to the observed changes of flagellar beating as well. Sensitivity to mechanical stimuli has been shown in sperm cells [66], but its relation to flagellar beating and the ability to fertilize eggs are not yet clear. Continuous contact with the micromotor could trigger such mechanoresponses and affect the performance of sperm-driven micromotors.

While it would be desirable to perform refined hydrodynamic computations exactly for the geometry of the micromotors used in this study, our lack of knowledge of the three-dimensional flagellar beat pattern, its interaction with the boundary surface, and last but not least the load response of the flagellar bending waves in response to hydrodynamic load, render such an approach at the moment unfeasible. Nonetheless, we can conclude already from our minimal model that the presence of a cargo container reduces normalized swimming speed, similar to the experimental observations for FRC and LRC micromotors.

## Conclusion

The regulation of flagellar propulsion is a complex mechanism that is still not fully understood. For the fabrication of sperm-based micromotors, an efficient propulsion is crucial. Typical degrees of freedom, like rolling and yawing, should be considered in the design of such micromotors. To a much greater degree, the flagellar load response and the hydrodynamic effect of additional cargo are responsible for changes in swimming speed. By fabricating two differently designed microcaps, we were able to constrain the rolling motion of cells. Yawing of cells was furthermore constrained by aligning the orientation of caps with an external magnetic field. These adjustments of the microcaps have a substantial effect on the flagellar beat frequency, which is responsible for the efficient propulsion of sperm-driven micromotors. Future optimizations of micromotor designs will have to take into account this flagellar load response, which appears to be at least as important for the net swimming speed as the direct hydrodynamic effect of additional hydrodynamic cargo. Thus, the biophysics of the flagellar beat and the hydrodynamics of swimming are equally important.

## Materials and methods

### Materials

The photoresist IP-Dip for 2-photon-polymerization was obtained from Nanoscribe GmbH. The sperm medium SP-TALP was prepared by dissolving 300 mg bovine serum albumin (Sigma) in 47.5 ml SP-TL (Caisson Labs). 2.5 ml of Na-pyruvate (Gibco) and $100 \, \upmu \hbox {l}$ of gentamycin were added and the solution was filtered sterile before storage at $4 ^{\circ }\,\hbox {C}$. Cryopreserved bovine semen samples were obtained from Masterrind GmbH.

### Fabrication of microcaps

Both types of microcaps were fabricated using 2-photon-photolithography (Photonic Professional GT, Nanoscribe GmbH). The 3D designs were prepared using the appropriate software (DeScribe, Nanoscribe GmbH). IP-Dip was dropcast on a glass quartz slide and polymerized at a laser power of 50%. After development for 13 min in mr-Dev 600 (microresist technology) samples were dried using critical point drying (EM CPD300, Leica). To allow for magnetic actuation as well as observation of rolling, the samples were then coated by electron beam deposituin (Plassys) with layers of 5 nm Ti, 10 nm Fe and 5 nm Ti at an angle of $60 ^{\circ }$ and a rate of 0.5 Å/s. During the process, samples were not rotated to achieve a one-sided deposition.

### SEM imaging and FIB cut

Imaging and FIB cut of microcaps were done using a NVision 40 (Zeiss). Samples were sputtered with 10 nm of Cr to improve conductivity and contrast. Imaging was performed at accelerating voltages of 2 kV (Fig. 1b) and 5 kV (Fig. 1c), and an aperture of $30 \, \upmu \text {m}$. To reveal the internal structure of the micromotors, FIB cuts were made with Ga+ ions of 30 kV and ion current of 3 nA, final polishing was done with 700 pA. No deposit was used because of the curved surface and the polymer matrix. The cutting resulted in smooth cut planes without significant curtaining for the structures.

### Sperm handling and sample preparation

Samples of a single bull were used for all experiments to avoid deviations between semen samples of different animals. Sperm samples were thawed at $37 ^{\circ }\,\hbox {C}$ and cleaned by centrifugation at 300 rcf for 5 min and resuspension in SP-TALP. A pellet swim-up was performed to select highly motile cells [67]. Sample chambers were prepared by separating two microscope cover slides by strips of parafilm resulting in channels of $150 \, \upmu \text {m}$ height [25]. Channels and microcaps were incubated with Pluronic F-127 for 30 min prior to experiments to avoid unspecific adhesion of biomolecules. Sperm samples were diluted to a final concentration of $1 \, \hbox {x} \, 10^{6} \, {\hbox {ml}}^{{-}1}$.

### Video recording

Videos of moving micromotors and sperm cells were recorded using an Axio Observer (Zeiss) with a heated microscope stage at $38 ^{\circ }\,\hbox {C}$ and an attached high-speed camera (Miro eX 2, Phantom). To observe the rolling of micromotors, videos were recorded at a framerate of 50 Hz. A framerate of 500 Hz and a phase-contrast filter were used to observe the rolling of sperm cells and to generate a high-contrast image of the cell’s flagellum for tracking of the flagellar dynamics.

### Analysis of flagellar beat frequency

Flagellar tracking was performed on high-speed recordings, using the ImageJ plugin SpermQ [50]. Image sequences of 2 s at framerate of 500 Hz were used for the analysis. The beat frequency was then extracted from the kymographs of the curvature angle.

### Measurement of micromotor rolling, speed and linearity

The one-sided deposition and the resulting shadow were used to track the rolling of micromotors. The reappearing shape was monitored, resulting in the frequency at which the micromotor is rolling around its longitudinal axis. VCL of micromotors was obtained by tracking the motors at a frequency of 500 Hz in the high-speed recordings, using the TrackMate plugin for ImageJ [68]. A moving average ($\pm 25$ points) was applied to the original trajectory to calculate VAP. The direct distance between the starting and endpoint of each trajectory was used to measure VSL. Linearity was calculated as LIN = VSL/VCL for trajectories with a length of 1500 frames (3.0 s).

### Measurement of cell rolling and speed

Blinking of the sperm head, generated by the rolling of the cell, was analyzed by measuring the pixel intensity along the cell trajectory. FFT was performed in Origin to find the frequency of sperm rolling. Since the sperm head is not visible inside the microcap, the curvature of the flagellum was used to determine the cells orientation in the case of captured cells. The frequency of reoccurring periods of low curvature was measured as it corresponds to the rolling frequency. The swimming speed and linearity of cells were measured in the same way as those of micromotors.

### Measurement of micromotor yawing

High-speed video microscopy recordings of sperm-driven micromotors were analyzed using custom-build Matlab software (The Mathworks, Inc.). In each frame, the bright phase halo around a microcap was determined in a region of interest ($100 \times 100$ pixels), using a percentile-based intensity threshold calculated from the typical size of the halo, followed by basic morphological operations to remove isolated pixels. The convex hull of the identified phase halo pixels defines a binary mask of the microcap. The centroid of this mask provides a robust estimate for the position of the sperm head in each frame, while its moments-of-inertia tensor provides the orientation of the long axis of the microcap. Given the time series of the orientation angle of the microcap, we computed its high-frequency component by subtracting a moving average. The periodogram of this component allows to extract the frequency and amplitude of yawing. We verified that the yawing frequency agrees with the beat frequency determined from flagellar tracking using the SpermQ software [50].

### Magnetic control of micromotors

The static magnetic field was generated by a permanent magnet, located at a distance of 20 mm from the sample. The field strength at this distance was measured to be 5 mT and 10 mT, depending on the strength of the magnet (Fig. S4 in supporting information).

### Statistical analysis

Two-sample t-tests were conducted using Origin to verify statistical significance of results. Probability values are given for a 0.05 significance level with Welch correction.

## Supplementary Information

Below is the link to the electronic supplementary material.

Supplementary material 1 (pdf 1512 KB)

Supplementary material 2 (avi 2802 KB)

Supplementary material 3 (avi 4628 KB)

Supplementary material 4 (avi 1968 KB)

Supplementary material 5 (avi 3651 KB)

Supplementary material 6 (avi 3601 KB)

## Data Availability

This manuscript has associated data in a data repository. [Authors’ comment: The data that support the findings of this study are available from the corresponding author, Mariana Medina-Sánchez, upon reasonable request.]
