# Supplementary material for: Micromotor-mediated sperm constrictions for improved swimming performance
Source: Eur Phys J E Soft Matter. 2021 May 11;44(5):67. doi: 10.1140/epje/s10189-021-00050-9 (PMC8113191; doi:10.1140/epje/s10189-021-00050-9)
Supplement: Supplementary file 1 — Supplementary material 1 (pdf 1512 KB) [file 10189_2021_50_MOESM1_ESM.pdf]

Figure S1: Representative kymographs from all samples.

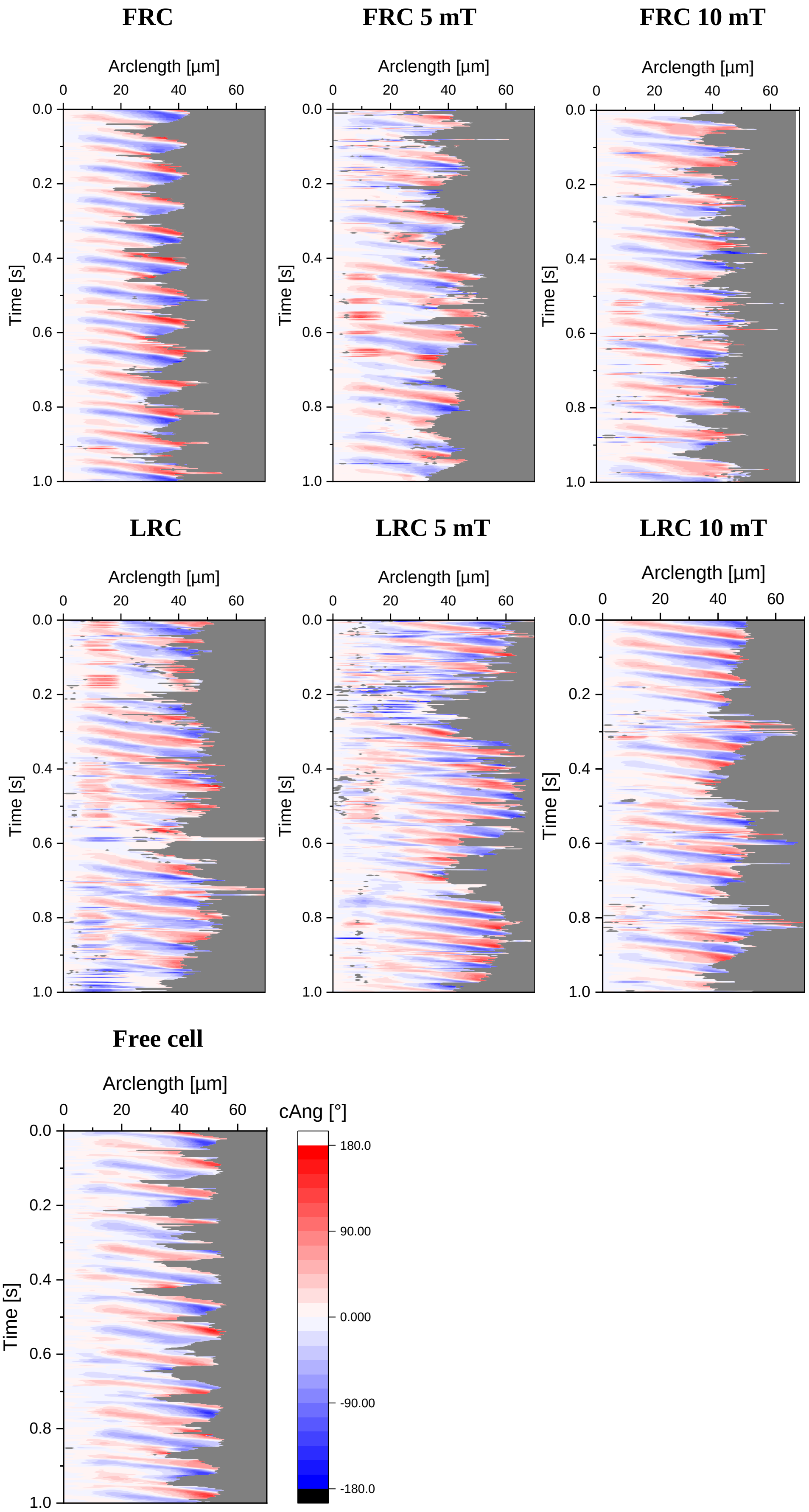

Table S1: Comparison of beat frequencies measured using SpermQ and the yawing frequency.

	FRC	FRC 5 mT	FRC 10 mT	LRC	LRC 5 mT	LRC 10 mT
Beat frequency ω_0 [Hz]	11.1 ± 1.9	10.3 ± 1.1	11.0 ± 0.7	16.6 ± 1.8	18.5 ± 2.6	16.5 ± 1.6
Yawing frequency [Hz]	11.1 ± 1.8	10.3 ± 1.0	10.0 ± 1.5	15.3 ± 2.0	18.7 ± 2.0	16.7 ± 2.7

Figure S2: Net swimming speed of a FRC micromotor measured over time with a periodically switched on magnetic field (B = 5 mT)

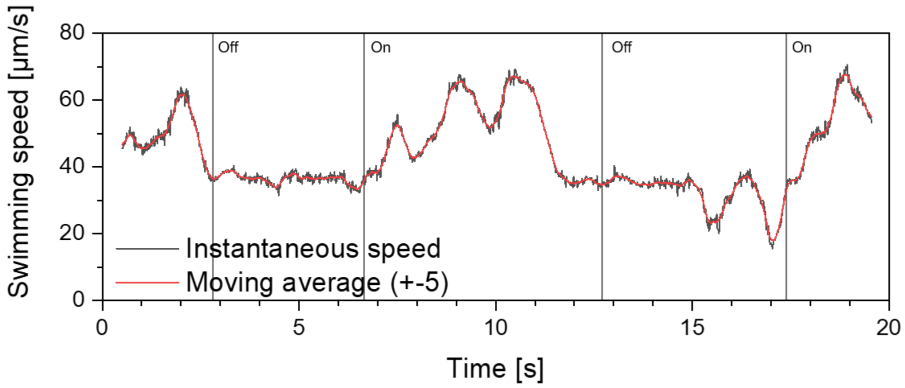

Figure S3: Beat frequency vs. cell rolling frequency

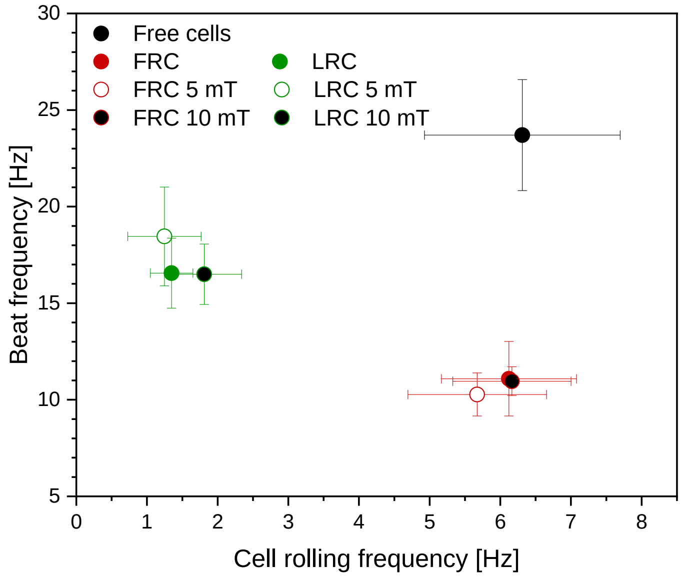

Figure S4: Magnetic field strength vs. distance from magnet

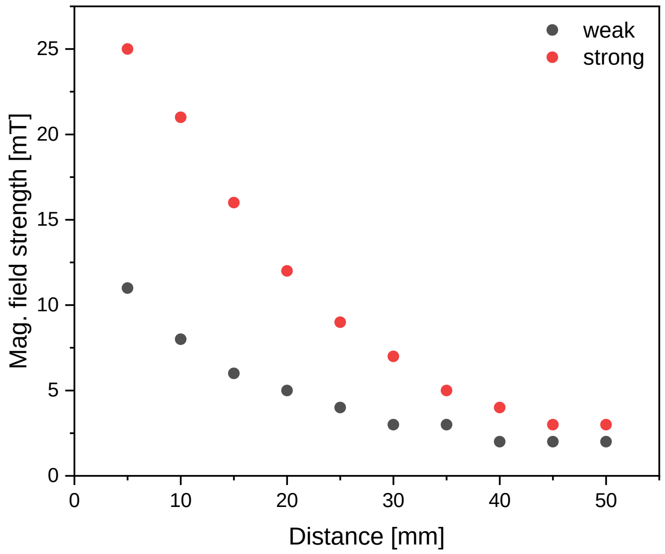

Movie S1: FRC and LRC micromotors moving with and without external magnetic field.

Movie S2: FRC micromotor moving while the magnetic field (5 mT) is periodically switched on and off.

Movie S3: Flagellar beat of a rolling sperm cell (slowed down 20x).

Movie S4: Flagellar beat of a rolling micromotor (slowed down 20x).

Movie S5: Rolling micromotor imaged by brightfield microscopy.
